# Stability of an aluminum salt-adjuvanted protein D-conjugated pneumococcal vaccine after exposure to subzero temperatures

**DOI:** 10.1080/21645515.2017.1421878

**Published:** 2018-02-12

**Authors:** Juliette Fortpied, Florence Wauters, Christelle Rochart, Philippe Hermand, Bernard Hoet, Nicolas Moniotte, Ivo Vojtek

**Affiliations:** GSK, Rixensart, Belgium

**Keywords:** pneumococcal conjugate vaccine, polysaccharide, alum, aluminum salt, shake test, vaccine thermosensitivity, cold chain, subzero temperature, freezing, vaccine thermostability

## Abstract

Accidental exposure of a vaccine containing an aluminum-salt adjuvant to temperatures below 0°C in the cold chain can lead to freeze damage. Our study evaluated the potential for freeze damage in a licensed aluminum-salt-containing protein-D-conjugated pneumococcal vaccine (PHiD-CV; Synflorix, GSK) in conditions that included static storage, single subzero-temperature excursions, and simulated air-freight transportation. Several parameters were assessed including freezing at subzero temperatures, aluminum-salt-particle size, antigen integrity and immunogenicity in the mouse. The suitability of the WHO's shake test for identifying freeze-damaged vaccines was also assessed. During subzero-temperature excursions, the mean temperatures at which PHiD-CV froze (−16.7°C to −18.1°C) appeared unaffected by the type of vaccine container (two-dose or four-dose vial, or single-dose syringe), vaccine batch, rotational agitation, or the rate of temperature decline (−0.5 to −10°C/hour). At constant subzero temperature and in simulated air-freight transportation, the freezing of PHiD-CV appeared to be promoted by vibration. At −5°C, no PHiD-CV sample froze in static storage (>1 month), whereas when subjected to vibration, a minority of samples froze (7/21, 33%) within 18 hours. At −8°C with vibration, nearly all (5/6, 83%) samples froze. In these vibration regimes, the shake test identified most samples that froze (10/12, 93%) except two in the −5°C regime. Nevertheless, PHiD-CV-antigen integrity appeared unaffected by freezing up to −20°C or by vibration. And although aluminum-salt-particle size was increased only by freezing at −20°C, PHiD-CV immunogenicity appeared only marginally affected by freezing at −20°C. Therefore, our study supports the use of the shake test to exclude freeze-damaged PHiD-CV in the field.

## Introduction

The cold chain is a common and critical requirement in vaccine distribution networks. Yet transient temperature changes from the stipulated storage requirement (temperature excursions) can occur; mainly because of events such as packaging malfunction during transportation, refrigerator failure or human error.^1-4^ In a significant number of cases, these temperature excursions represent temperature drops below the stipulated storage temperature range and sometimes may fall below 0°C, leading to potential freeze damage. Freeze damage to a vaccine can decrease the vaccine's immunogenicity and increase its potential to cause local reactions.^3-9^

Freeze damage in aluminum-salt adjuvanted vaccines can be detected using the shake test; a simple straightforward test developed and validated by the World Health Organization (WHO) for use by medical practitioner in the field.^10^ The test works on the basis that when a vaccine goes through a freeze-thaw cycle, the aluminum-salt particles agglomerate and form a precipitate that rapidly sediments out of solution. Although a number of aluminum-salt adjuvanted vaccines have been evaluated,^3^^,^^10^ the effects of freeze damage and the reliability of freeze-damage detection by this shake test have not been evaluated for pneumococcal conjugate vaccines.

The licensed protein-D-conjugated pneumococcal vaccine (PHiD-CV; Synflorix, GSK) is a vaccine for the prevention of pneumococcal diseases such as septicemia, meningitis, pneumonia and acute otitis media in children aged six weeks to five years. It contains capsule polysaccharides from 10 of the most common types of *Streptococcus pneumoniae* bacteria (1, 4, 5, 6B, 7F, 9V, 14, 18C, 19F and 23F) conjugated to carrier proteins. It also contains aluminum phosphate as an adjuvant. Its recommended storage temperature is +2 to +8°C.^11^

The objectives of our study were (i) to evaluate the freezing characteristics of PHiD-CV in conditions that included static storage and simulated air-freight transportation, and (ii) to evaluate the relationships between these characteristics of vaccine freezing, shake-test outcomes, and vaccine potency and immunogenicity.

## Results

### Characterization of vaccine freezing

Two experimental scenarios were considered in the characterization of vaccine freezing: (i) exposure to a decline in temperature (from +4°C to −20°C) over a relatively short period of time (within 48 hours; i.e. a temperature excursion) without or with rotational agitation; and (ii) exposure to a constant subzero temperature without or with vibration, simulating the kinetic conditions of a transport container within an aircraft. The point at which freezing occurred was determined as the temperature recorded on the surface of the vaccine container just prior to the transient rise in temperature corresponding to the exothermic release of energy with the transition from liquid to solid of the water in the vaccine. Freezing was confirmed by visual inspection of the vaccine. An indirect assessment of vaccine freezing was performed using the WHO shake test, whereby at room temperature and after agitation, a freeze-damaged vaccine sample presents as a rapidly sedimenting granular white precipitate in a clear solution, in contrast to a negative control sample which presents as an opaque solution.

### Vaccines in different types of container exposed to single temperature excursions

In the first experiment, PHiD-CV samples in three different types of container (2-dose vial, 4-dose vial and 1-dose syringe) were exposed to a temperature decline from 4°C to −20°C with a rate of −0.5°C/hour. Samples in 2-dose vials were also exposed to temperature declines of −1.0°C/hour and −10°C/hour, and to a temperature decline of −0.5°C/hour combined with rotational agitation (orbital shaker at 600 rpm).

With the temperature decline of −0.5°C/hour and without agitation, all 24 PHiD-CV samples froze in the 2-dose vials and all 8 samples froze in the 4-dose vials. Most (6/7) but not all samples froze in the syringes. For the 2-dose vials and with the temperature declines of −1.0°C/hour and −10°C/hour without agitation, all samples froze (8/8 and 9/9, respectively). For the 2-dose vials and with an exposure to a temperature decline of −0.5°C/hour combined with rotational agitation, most (6/8) but not all samples froze.

**Figure 1.**
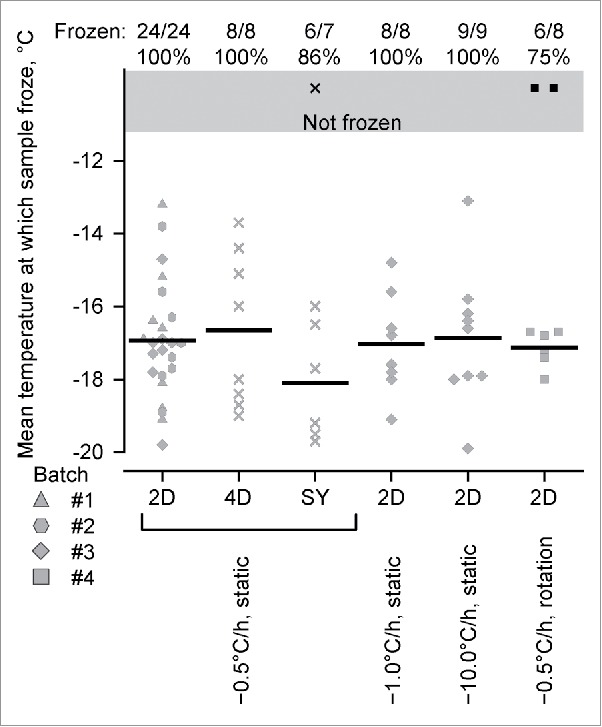
The characterization of PHiD-CV freezing with exposure to a single subzero temperature excursion. The temperatures at which PHiD-CV samples froze in two-dose vials (2D), four-dose vials (4D) and syringes (SY), in regimes in which the temperature was lowered by either 0.5°C/hour, 1.0°C/hour, or 10°C/hour from +4°C to −20°C. In the regime with rotational agitation (rotation), PHiD-CV samples were attached to an orbital shaker set at 600 rpm. Horizontal black bars indicate mean values and symbols indicate individual values, differently shaped symbols indicate different batches of PHiD-CV samples in the two-dose vials, and the diagonal crosses indicate PHiD-CV samples in the other types of container. The proportions of samples that froze are indicated above the graphs, and the symbols shaded in black represent those samples that did not freeze and were not included in the calculation of the mean temperatures of freezing.

Among the 61 PHiD-CV samples that froze, the overall mean temperature at which freezing occurred was −17.0°C (standard deviation, 1.60), the highest individual temperature at which freezing occurred was −13.1°C, and the mean temperatures at which freezing occurred in each group ranged from −16.7°C to −18.1°C (Fig. 1). With exposure to a temperature decline of −0.5°C/hour, the mean temperature at which freezing occurred in the 2-dose vials was −16.9°C (N = 24), and similar between the three different PHiD-CV batches (≤0.3°C difference in mean freezing temperatures, where each batch included 8 samples). The mean temperatures at which freezing occurred were similar between samples in the 2-dose vials, 4-dose vials (−16.7°C, N = 8), and syringes (−18.1°C, N = 6). The mean temperatures at which freezing occurred in the 2-dose vials with exposure to −1.0°C/hour and −10°C/hour temperature declines (−17.0°C, N = 8 and −16.9°C, N = 9, respectively), or to −0.5°C/hour temperature decline with rotational agitation (−17.1°C, N = 6) were similar to that with the static exposure to a −0.5°C/hour temperature decline (−16.9°C, N = 24).

### Vaccines exposed to constant subzero temperatures in transport packages

**Figure 2.**
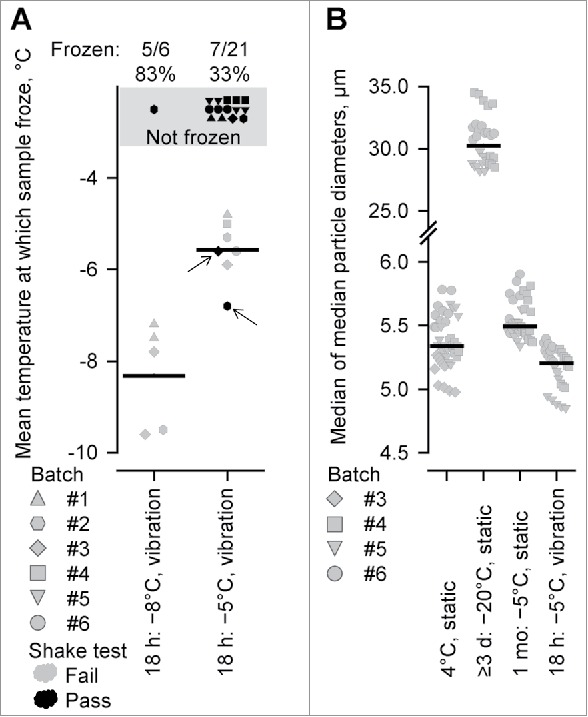
The characterization of PHiD-CV freezing with exposure to constant subzero temperatures and conditions simulating air-freight transportation. (A) The temperatures at which PHiD-CV samples froze in the two-dose vials in conditions of simulated air-freight transportation. Samples were placed for 18 hours in refrigerated transport packages, subjected to a standardized vibration protocol (power spectral density of 0–0.01 G^2^/Hz at a frequency of 0–300 Hz), and in which the internal temperature was relatively constant (−5°C and −8°C). (B) Median particle diameters of PHiD-CV samples in the two-dose vials in regimes including static exposure at +4°C (storage), 1 month static exposure at −5°C, ≥3 days static exposure at −20°C, and the −5°C vibration regime described in (A). Horizontal black bars indicate mean values in (A) and median values in (B). In (A) and (B), symbols indicate individual values, and differently shaped symbols indicate different batches of PHiD-CV samples in the two-dose vials. In (A), the proportions of samples that froze are indicated above the graphs, and the symbols shaded in black and superimposed on the gray background represent those samples that did not freeze and were not included in the calculation of the mean temperatures of freezing. In (A), samples in gray failed the shake test (indicating “freezing”), samples in black passed the shake test (indicating “no freezing”). Arrows show samples that were frozen but passed the shake test.

In the second experiment, and to simulate air-freight transportation conditions, PHiD-CV samples (in 2-dose vials) were placed for 18 hours in refrigerated transport packages, which were subjected to a vibration protocol (power spectral density of 0–0.01 G^2^/Hz at frequency of 0–300 Hz).^12^ Two temperature regimes were evaluated in which the targeted internal temperature was either −8°C or −5°C, and were obtained by inserting into the packages, cold blocks (pre-cooled at −20°C) that equilibrated the transport package to a temperature below the targeted temperatures (ca. 2.5°C below) before the samples were introduced. With the −8°C vibration regime, 83% (5/6) of the samples froze, whereas with the −5°C vibration regime, 33% (7/21) of the samples froze (Fig. 2A). The mean incubation period to freezing was 12.3 hours in the −8°C vibration regime and 9.9 hours in the −5°C vibration regime, and the shortest period to freezing was 4 hours (Supplementary Fig. 1A). The temperatures at which the samples froze in either regime reflected the actual temperatures of the vaccines in the refrigerated transport package at those time points, and these mean temperatures were −8.3°C in the −8°C vibration regime and −5.7°C in the −5°C vibration regime.

All 5 PHiD-CV samples that froze in the −8°C vibration regime and 5/7 samples that froze in the −5°C vibration regime also failed the shake test (overall shake-test failure: 10/12, 83%; Fig. 2A). In each of those 10 samples that failed the shake test, the precipitate was flocculated in appearance (and not granular as observed with the control samples that had been frozen at −20°C). The two samples that passed the shake test also displayed floccular precipitates, but the sedimentation rates were lower than that in control −20°C samples.

Given that the basis for a positive shake test is the agglomeration of aluminum-salt particles, the outcome of the shake test and its relationship to vaccine-particle size (i.e. aluminum-salt particle with adsorbed antigens) was evaluated further in the 18 hour/−5°C vibration regime, in comparison with a −5°C static regime of 1-month duration and two control static regimes (routine storage at +4°C and ≥3 days at −20°C). As expected, in the +4°C static regime, no (0/20) PHiD-CV sample froze or failed the shake test, whereas after ≥3-days in −20°C static regime, all (20/20) samples froze and failed the shake test with the appearance of a granular precipitate. In contrast to the −5°C vibration regime where 7/21 froze and 5/7 frozen samples failed the shake test, no (0/20) sample froze or failed the shake test after 1 month static exposure at −5°C.

Vaccine-particle sizes were measured by laser diffraction static light scattering (LD-SLS) and in a given PHiD-CV sample, the distribution of vaccine-particle sizes was captured by calculating the median, 10^th^ and 90^th^ percentiles of particle diameters (Fig. 2B and Supplementary Fig. 1B and 1C). In the +4°C static regime (in which no sample failed the shake test), the median of the median diameters was 5.3 µm (45 samples from 4 vaccine batches; Fig. 2B), and the medians of the 10^th^ and 90^th^ percentiles were 2.9 and 9.6 µm, respectively (Supplementary Fig. 1B and 1C). In the −20°C regime (in which all samples failed the shake test), the median particle size was 6-fold larger than in the +4°C static regime: the median of the median diameters was 30.2 µm (30 samples from 3 vaccine batches; Fig. 2B) and the median of the 10^th^ and 90^th^ percentiles were 9.6 and 66.0 µm, respectively (Supplementary Fig. 1B and 1C). Therefore in the −20°C regime, the large particle size corresponded well with the granular precipitate observed after the shake test.

In the −5°C static regime (in which no PHiD-CV sample failed the shake test), the median particle size was similar to that in the +4°C static regime: the median of the median diameters was 5.5 µm (35 samples from 3 vaccine batches; Fig. 2B), and the medians of the 10^th^ and 90^th^ percentiles were 3.0 and 10.0 µm, respectively (Supplementary Fig. 1B and 1C). After 18 hours exposure to vibration at −5°C, the particle sizes were similar to those in the two static regimes (+4°C and −5°C): the median of the median diameters was 5.2 µm (30 samples from 3 vaccine batches; Fig. 2B) and the median of the 10^th^ and 90^th^ percentiles were 2.8 and 9.4 µm, respectively (Supplementary Fig. 1B and 1C). Therefore, although a fail result in the shake-test was relatively infrequent in the −5°C vibration regime, there was no evidence to suggest that the flocculated precipitate observed after the shake test was derived from the formation of relatively large particles.

### Effect of constant subzero temperature on vaccine potency and vaccine immunogenicity

**Figure 3.**
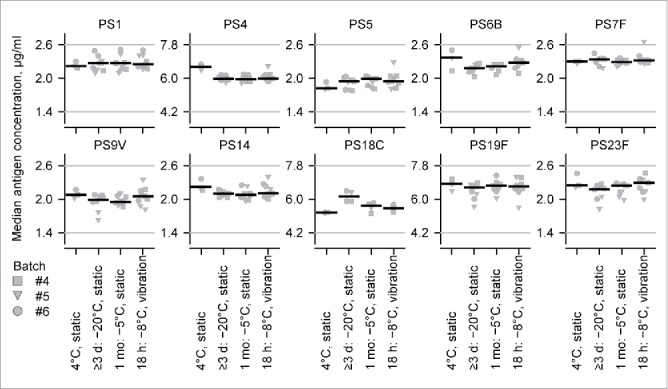
The effect of different subzero regimes on polysaccharide (PS) antigen concentrations. Antigen concentrations by *Streptococcus pneumoniae* serotype in PHiD-CV samples in the two-dose vials in regimes including static exposure at +4°C (storage), 1 month static exposure at −5°C, ≥3 days static exposure at −20°C, and 18 hours in refrigerated (−8°C) transport packages, subjected to a standardized vibration protocol (power spectral density of 0–0.01 G^2^/Hz at a frequency of 0–300 Hz). Horizontal black bars indicate the median values, symbols indicate individual values, and differently shaped symbols indicate different batches of PHiD-CV samples. Horizontal gray lines represent the quality-control minimum and maximum concentration limits for vaccine release.

The potency of PHiD-CV in terms of antigen concentrations and potential antigenicity (i.e. antigen integrity) was evaluated by rate nephelometry (the measurement of the change of light scattering caused by the formation of complexes of antigen and cognate antibody; Fig. 3). Three subzero regimes were evaluated (1 month static at −5°C, ≥3 days static at −20°C and 18 hours with vibration at −8°C), in addition to the control regime of static at +4°C. For each of the subzero regimes, 9 samples (3 samples × 3 batches) were evaluated for each antigen except for polysaccharide 6B (range: 6–7 samples) and polysaccharide 18C (4 samples) where some samples were lost due to breakage. For the +4°C regime, 3 samples (from 3 batches) were evaluated.

No PHiD-CV sample evaluated contained antigens at a concentration below the minimum quality-control release concentration (Fig. 3). The difference in median concentrations between all four regimes for a given antigen type was typically <10% and was at most 16% (polysaccharide 18C; −20°C versus +4°C). Therefore the potency of PHiD-CV (i.e. antigen concentrations) appeared unaffected by freezing up to −20°C or by vibration.

**Figure 4.**
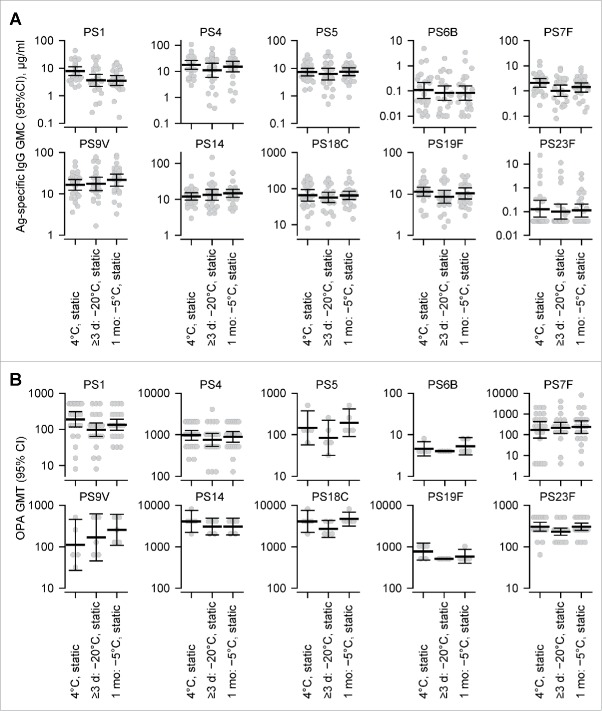
The effect of different subzero regimes on PHiD-CV immunogenicity in mice. (A) Antibody concentrations and (B) opsonophagocytosis-assay (OPA) titers by *Streptococcus pneumoniae* serotype from murine serum samples taken 14 days after vaccination, where each mouse received three doses (two-weeks apart) of a given treated PHiD-CV sample. The PHiD-CV samples (two-dose vials) had been subjected to regimes including static exposure at +4°C (storage), 1 month static exposure at −5°C, and ≥3 days static exposure at −20°C. Horizontal wide black bars indicate the geometric mean concentrations (GMCs) or geometric mean titers (GMTs), and symbols indicate individual values. Black error bars indicate 95% confidence intervals (95% CIs).

The immunogenicity of PHiD-CV was evaluated in a mouse model by determining antigen-specific antibody (IgG) concentrations and antibody function (opsonophagocytosis-assay [OPA] titers) in serum samples 14 days after the injection of three doses of PHiD-CV (14-days apart; Fig. 4). Two subzero regimes were evaluated (1 month static at −5°C and ≥3 days static at −20°C), in addition to the control regime of static storage at +4°C. For antibody concentrations and OPA titers, 24–25 samples per regime were evaluated, except for OPA titers against polysaccharides 5, 6B, 9V, 14, 18C and 19F for which 5 pools of 5 samples per regime were evaluated.

In the −5°C regime, only one of the antigen-specific IgG geometric mean concentrations (GMCs) was >2-fold lower than the respective concentrations in the +4°C regime (Fig. 4A), and that was for polysaccharide 1 (2.2-fold lower). In the −20°C regime, only two antigen-specific IgG GMCs were more than 2-fold lower than the respective GMCs in the +4°C regime, and those were specific for polysaccharide 1 and for polysaccharide 7F (2.1-fold lower in both cases). In both the −5 and −20°C regimes, none of the antigen-specific OPA geometric mean titers (GMTs) was >2-fold lower than the respective GMTs in the +4°C regime (Fig. 4B). Therefore, the immunogenicity of PHiD-CV appeared only marginally and partially affected by one month static storage at −5°C or by freezing at −20°C.

## Discussion

PHiD-CV is a licensed vaccine for the prevention of pneumococcal diseases in children. It contains 10 antigens adsorbed onto aluminum-salt particles and requires transportation and storage to operate at temperatures in the range of +2 to +8°C.^11^ Our study evaluated the potential for freeze damage to PHiD-CV as a consequence of accidental exposure to subzero temperatures, and the suitability of the WHO's shake test for identifying in the field such freeze damage through measuring the sedimentation of aluminum-salt particles.

With exposure to subzero temperatures, the tendency of the aluminum-salt particles to agglomerate is considered to increase with the formation of ice crystals.^3^^,^^13-15^ Also, rotational agitation or vibration, or differences in the internal surface of the vaccine container could potentially promote ice-crystal formation. Conversely, rotational agitation or vibration could reduce the tendency for aluminum-salt particle sedimentation and hence reduce the tendency for the aluminum-salt particles to agglomerate.

During single subzero temperature excursions without or with rotational agitation, 61/64 PHiD-CV samples froze when the temperature declined to at least −13°C (and on average down to −16.7°C to −18.1°C), and at rates of decline between −0.5°C/hour and −10°C/hour. The failure to detect freezing in 3/64 samples may have been due to −20°C not being sufficiently low enough to capture all freezing events. Nevertheless, the temperature at which PHiD-CV froze appeared unaffected by the type of vaccine container (two-dose vial, four-dose vial or one-dose syringe), by PHiD-CV batch, by rotational agitation, or by the rate of temperature decline (−0.5 to −10°C/hour). Even though the first signs of sedimentation of an aluminum-salt adjuvant have been observed within 15 minutes of the adjuvant being left still,^13^ the result suggested that degree of vaccine sedimentation, which would have been inversely proportional to the rate of temperature decline, was unlikely to have affected PHiD-CV freezing.

At a constant subzero temperature in conditions that simulated air-freight transportation, the freezing of PHiD-CV appeared to be promoted by vibration. At a temperature threshold of −5°C, no PHiD-CV sample froze in static storage (over one month), whereas when subjected to vibration, some samples (33%, 7/21) froze within a period of 18 hours. Similar observations have been made with an aluminum-salt adjuvanted hepatitis B vaccine: that vaccine failed to freeze after 72 hours static exposure at −6°C, whereas the vaccine froze within 3 hours when the exposure at −6°C was combined with rotational agitation by orbital shaker (ca. 60–120 rpm).^6^ However, unlike the hepatitis B vaccine, for those PHiD-CV samples that froze, the mean periods of time to freezing were longer; 9.9 hours and 12.3 hours at −5°C and −8°C (where 83% [5/6] samples froze), respectively, which may reflect differences in the nature of the formulations or the design of the experiments. The period of time to freezing also suggests that the vibration protocol was effective at promoting PHiD-CV freezing, in contrast to the rotational-agitation protocol used in the temperature excursion regime (−0.5°C/hour). It could be estimated that if the PHiD-CV samples in the temperature excursion regime (−0.5°C/hour) were subjected to the vibration protocol instead of rotational agitation then the temperature at which the vaccine would freeze would be ca. −14°C (from −8°C, the temperature would have declined by ca. 6°C within 12.3 hours) and higher than the observed −17.1°C. Therefore the apparent effectiveness of the vibration protocol in promoting PHiD-CV freezing may relate to the (lower or higher) degree of agitation or turbulence in the vaccine container.

The shake test correctly identified most PHiD-CV samples that froze, except two samples in the −5°C/vibration regime. Furthermore, the appearance of the precipitates in the −5°C/−8°C/vibration regimes were different from those in the −20°C static regime after the shake test (i.e. floccular versus granular); and PHiD-CV- (aluminum-salt-) particle agglomeration in terms of particle size was only suggested from the −20°C regime. Hence, the physicochemical interactions between aluminum-salt particles may have been weaker or disrupted in −5°C/−8°C vibration regimes in contrast to a static regime at a lower temperature (i.e. single temperature excursion or ≥3 days at −20°C). Other factors unrelated to vibration may have also affected particle size. In the evaluation of the aluminum-salt adjuvanted hepatitis B vaccine, a lower temperature (−20°C versus −6°C and −10°C) and the duration of the exposure to that temperature (which led to a reduction in the surface charge of the particles and thus presumably lowered the repulsion between particles) were associated with increases in particle size.^6^ Interestingly, in the shake-test validation study,^10^ partially frozen or “slushy frozen” vaccines of various types were prepared by short exposure to −10°C. All 18 samples evaluated passed the shake test (i.e. indicating not frozen), and the particle sizes (by phase contrast) in these partially frozen vaccines were similar to those of the unfrozen vaccines. This suggested that the frozen PHiD-CV samples in the −5°C/−8°C/vibration regimes and slushy frozen vaccines represented borderline cases for the shake test whereby permanent changes to the physicochemical properties of the vaccine were negligible.

In the constant subzero-temperature regimes evaluated in our study, the potency of PHiD-CV, in terms of antigen concentrations and potential antigenicity, appeared unaffected by freezing or by vibration, and the immunogenicity of PHiD-CV in the mouse model appeared only marginally and partially affected by one month static storage at −5°C or by freezing at −20°C. The physicochemical observations concur with the results from a study of the thermostability of polysaccharide components of PHiD-CV, where the size, integrity and pH stability of each of the 10 conjugated polysaccharides appeared unaffected by storage at −20°C.^16^ Nevertheless, in our study, subtle and undetected changes to the properties of polysaccharide 1 and 7F antigens may have occurred at −20°C and may have affected their immunogenicities. Alternatively, the observed changes in immunogenicities were within the range of variability of the mouse model, because from GSK's experience in the vaccine's development, a GMC or GMT difference in the mouse model of <3-fold is considered unlikely to be biologically relevant (data not shown).

Overall, the results from our study suggest that for the borderline cases of PHiD-CV samples partially frozen to PHiD-CV samples completely frozen after a single exposure to −20°C, the shake test will tend to reject samples in which the immunogenicities and physicochemical properties of the antigens are not compromised, even though aluminum-salt particles show signs of agglomeration. Hence the shake test represents a relatively conservative and useful method to exclude potentially freeze-damaged PHiD-CV samples in the field.

## Materials and methods

### Vaccine, antigens and antibodies

PHiD-CV individual un-conjugated polysaccharide antigens (1, 4, 5, 6B, 7F, 9V, 14, 18C, 19F and 23F) and corresponding serotype-specific antibodies (for the ELISA) were from GSK. One human dose of PHiD-CV contains a 0.5-ml suspension comprising 1 µg each of polysaccharide antigens 1, 5, 6B, 7F, 9V, 14 and 23F and 3 µg each of polysaccharide antigens 4, 18C and 19F. In the vaccine, polysaccharide antigens 1, 4, 5, 6B, 7F, 9V, 14 and 23F are conjugated to non-typeable *Haemophilus influenzae* (NTHi) protein D, polysaccharide 18C is conjugated to tetanus toxoid, and polysaccharide 19F is conjugated to diphtheria toxoid. For the experiments with the two-dose vials, six different batches of PHiD-CV were used (as indicated in the figure legends).

### Subzero temperature regimes and temperature monitoring

Controlled temperature declines were performed using a climate chamber (MK53, Binder). Rotational agitation was applied by placing an orbital shaker (M72 Minishaker, IKA) set at 600 rpm with vaccine containers attached, into the climate chamber.

A standardized protocol was used to simulate air-freight transportation.^12^ The transport package was cooled by the addition of cool packs frozen at −20°C (type E−6 or a mixture of types E-6 and E-10 to target a final temperature of −5°C or −8°C in the transport package, respectively; PCM Products Ltd.). The internal temperature was equilibrated over a period of 4 hours to a temperature approximately 2.5°C below the target temperature (−5°C or −8°C), after which the samples from +4°C storage were placed in the package (at time = 0 hours). Under those experimental conditions, the internal temperature around the target temperature was relatively stable for 48 hours. The vaccine samples were incubated for 4 hours in a static condition for the temperature in the vaccine containers to equilibrate. After 4 hours, the transport container was attached to a platform moving in horizontal and vertical directions (maximum of 6.4 cm elevation) and vibration was applied for a period of 18 hours at a power spectral density of 0–0.01 G^2^/Hz and a frequency of 0–300 Hz.

The temperature of a vaccine sample was recorded using a monitor (DX2030-S5H701148, Yokogawa) and a thermocouple probe attached to the outside of the vaccine container by thermal paste (340 heat sink compound, Dow Corning, ref. 01015443).

### WHO shake test

The shake test was performed in accordance with the standard protocol.^3^^,^^10^ Briefly, the contents of tested vials were gently mixed on a 360° rotate axis with 15 cycles at 30 rpm and sedimentation was recorded photographically (Axio Vision 4.6 Zeiss 002–16071) at one-minute intervals over 10 minutes. The static incubation of vaccine samples at +4°C (routine storage) and −20°C (≥3 days followed by thawing at room temperature) represented negative and positive control regimes, respectively. Test failure was demonstrated when the test sample sedimented at an equivalent or faster rate than the positive control samples (≥3 days, −20°C) regime.

### Particle size measurement

The size distribution of the vaccine particles was determined by laser diffraction using a Hydro2000S wet sample dispersion unit and Mastersizer 2000 (Malvern Instruments Ltd). The parameters were: for wavelengths, 633 nm and 455 nm; for obscuration, 5 to 7%; for recirculation speed, 2000 rpm; and the dispersant was 150 mM NaCl. Mie theory was applied with the following optical parameters: the real refractive index of the particles was 1.65; and the imaginary refractive index of the particles was 0.01.

### Rate nephelometry assay

The vaccine sample (in triplicate) was incubated with a protease (trypsin [Sigma ref. T9316] or pronase E [Merck ref. 1.074.330005 and 1.074.330001], depending on the serotype). Then the vaccine sample was centrifuged to remove the aluminum-salt particles. In the rate nephelometer (Immage 800, Beckman Coulter), the supernatant and the serotype-specific antibody were mixed in the cuvette and incubated for 5 minutes at 37°C. Scattered-light measurements (from a 670 nm wavelength source at 90° to the detector) were recorded every 5 seconds. The concentration of the antigen was based on the kinetics of the antigen/antibody complex formation, manifested by changes in light-scatter signal over time and was calculated with reference to a standard curve generated using vaccine samples with known concentrations of antigens.

### Husbandry and vaccination procedures in mice

The husbandry and vaccine immunogenicity evaluations on mice were ethically reviewed and carried out in accordance with European Directive 2010/63/EU and the GlaxoSmithKline Biologicals SA policy on the Care, Welfare and Treatment of Animals. Four-week old female Balb/c mice (purchased from Harlan Horst) were group housed (10 mice per cage) in type-3 cages under day/night cycles of 12 hours (6am–6pm) and were provided with dry food and filtered water *ad libitum*. The vaccine (50 µl per dose [1/10 human dose]) was administered into the left *gastrocnemius* muscle without anesthesia in a schedule of three doses two-weeks apart. Blood samples were collected 14 days after the third dose from which serum samples were prepared.

### ELISA to measure the levels of serum IgG to each capsule polysaccharide

Microtiter 96-well ELISA plates (MAXISORP, Thermo Scientific) were coated 2 hours at 37°C with 100 µl per well of polysaccharide in phosphate buffer saline (PBS) at 2.5 µg/ml (serotype 1), 5 µg/ml (serotypes 4, 5, 6B, 7F, 9V and 14), 10 µg/ml (serotype 23F) and 40 µg/ml (serotypes 18C, 19F). Plates were washed three times with 0.05% Tween 20 in 150 mM NaCl. Serum samples were diluted in an equal volume of 0.05% Tween 20 in PBS containing 1 mg/ml C-polysaccharides (CPS) and incubated 1 hour at 37°C to neutralize non-specific antibodies. Serial two-fold dilutions of the CPS/serum samples were added into the ELISA plate and incubated for 30 minutes at room temperature. Peroxidase-conjugated goat anti-mouse IgG antibodies (1/5000 dilution; ref. 115-035-033, Jackson Laboratory), *O*-phenylenediamine dihydrochloride and H_2_O_2_ were used to reveal polysaccharide-specific IgG binding. The individual IgG concentrations (expressed as µg/ml) were calculated using the 4-parameter method using the Soft Max Pro software and based on optical density (490 nm) readings.

### Opsonophagocytosis assay (OPA)

The serum samples were heated at 56°C (to inactivate the complement) and then diluted in a two-fold series in 25 µl Hank's buffered salt solution containing 3% bovine serum albumin in a 96-well round bottom plate. Twenty-five µl of a suspension of differentiated HL-60 cells (parental promyelocytic HL-60 cells were differentiated into neutrophils by incubation with N,N dimethylformamide), pneumococci and neonatal rabbit complement, were mixed in a 4/2/2 ratio (serotypes 1 and 6B) or a 4/2/1 ratio (serotypes 4, 5, 7F, 9V, 14, 18C, 19F and 23F). The plates were incubated for 2 hours at 37°C (with agitation to promote phagocytosis). A 20 μl aliquot of each well was then transferred into the corresponding well of a 96-well flat bottom microplate. Fifty µl of 0.9% agar in Todd-Hewitt broth were added twice into each well. After overnight incubation at 37°C, the pneumococcal colonies were counted using an automated image analysis system (Axiovision). The mean number of colony forming units of eight wells containing bacteria without any serum was used as the positive control for the calculation of the bacteria killing activity of a serum sample. The OPA titers were expressed as the dilution of serum inducing 50% bacterial death.

### Statistics

For temperatures, means were calculated. For vaccine-particle sizes, the 10^th^, 50^th^ (median) and 90^th^ percentiles of the particle sizes were calculated in each sample. Then for all samples within each treatment group, the three medians were calculated for the 10^th^, 50^th^ and 90^th^ percentiles, respectively. For antigen concentrations, medians were calculated. For antibody concentrations and OPA titers, geometric means and 95% confidence intervals were derived by back transformation of means and Student *t* 95% confidence intervals calculated on log_10_-transformed data. The sample sizes for the measurements of antibody concentrations and OPA titers were powered on the basis that ≥3 fold differences between treatments were potentially biologically relevant.

## Supplementary Material

KHVI_A_1421878_Supplemental.pdf

## Abbreviations

OPA: opsonophagocytosis assay
PHiD-CV: protein D-conjugated pneumococcal vaccine
