# Supplementary material for: Stability of an aluminum salt-adjuvanted protein D-conjugated pneumococcal vaccine after exposure to subzero temperatures
Source: Hum Vaccin Immunother. 2018 Feb 12;14(5):1243–50. doi: 10.1080/21645515.2017.1421878 (PMC5989897; doi:10.1080/21645515.2017.1421878)
Supplement: KHVI_A_1421878_Supplemental.pdf [file khvi-14-05-1421878-s001.pdf]

Supplementary Figure 1

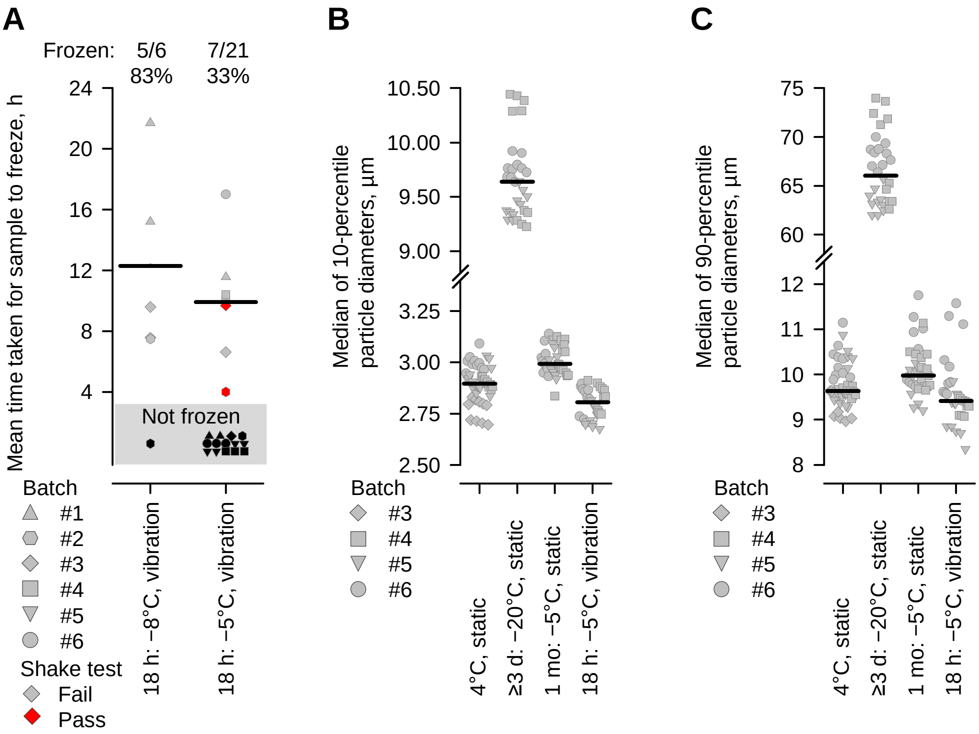

Supplementary Figure 1: Additional characterization of PHiD-CV freezing with exposure to constant subzero temperatures and conditions simulating air-freight transportation. (A) The time period to vaccine freezing of PHiD-CV samples in the two-dose vials (shown in Figure 2A), placed for 18 hours in refrigerated transport packages, subjected to a standardized vibration protocol (power spectral density of 0–0.01 G2/Hz at a frequency of 0–300 Hz), and in which the internal temperature was relatively constant (−5°C and −8°C). (B) 10th percentile particle diameters and (C) 90th percentile particle diameters of PHiD-CV samples in the two-dose vials (shown in Figure 2B) in regimes including static exposure at +4°C (storage), 1 month static exposure at −5°C, ≥3 days static exposure at −20°C, and the −5°C vibration regime. In (A), (B) and (C), horizontal black bars indicate the median values; symbols indicate individual values, and differently shaped symbols indicate different batches of PHiD-CV samples. In (A), the proportions of samples that froze are indicated above the graph, and the symbols shaded in black represent those samples that did not freeze and were not included in the calculation of the mean time period to freezing or the shake test. In (A), symbols shaded in gray and red represent those samples that failed (indicated freezing) and passed (indicated no freezing) the shake test, respectively.
